# Determinants of geographic variation in the incidence of adult nonmalignant meningioma in the United States, 2010–2019

**DOI:** 10.1002/cncr.70042

**Published:** 2025-09-11

**Authors:** Diana R. Withrow, Joseph Boyle, Martha S. Linet, Christine A. Pittman Ballard, Gino Cioffi, Carol Kruchko, Donald L. Miller, Valentina I. Petkov, Mackenzie Price, Kristin Waite, Quinn T. Ostrom, Jill S. Barnholtz‐Sloan

**Affiliations:** ^1^ Department of Health Services Research and Policy London School of Hygiene and Tropical Medicine London UK; ^2^ Massey Comprehensive Cancer Center Virginia Commonwealth University Richmond Virginia USA; ^3^ Division of Cancer Epidemiology and Genetics National Cancer Institute National Institutes of Health Rockville Maryland USA; ^4^ Department of Neurosurgery Duke University School of Medicine Durham North Carolina USA; ^5^ Central Brain Tumor Registry of the United States Hinsdale Illinois USA; ^6^ Center for Devices and Radiological Health US Food and Drug Administration Silver Spring Maryland USA; ^7^ Division of Cancer Control and Population Sciences Surveillance Research Program National Cancer Institute National Institutes of Health Rockville Maryland USA; ^8^ The Preston Robert Tisch Brain Tumor Center Duke University School of Medicine Durham North Carolina USA; ^9^ Duke Cancer Institute Duke University Medical Center Durham North Carolina USA; ^10^ Center for Biomedical Informatics and Information Technology National Cancer Institute National Institutes of Health Rockville Maryland USA

**Keywords:** brain tumor, detection, diagnosis, geography, incidence, meningioma

## Abstract

**Background:**

US incidence rates of nonmalignant brain tumors are 3‐fold higher in highest versus lowest incidence states. A county‐level analysis was conducted to assess whether geographic variation in nonmalignant meningioma (NMM) incidence is related to demographics, cancer registry, health care, and other factors.

**Methods:**

Age‐adjusted incidence rates of NMM in US counties during 2010–2019 were modeled with data from the Central Brain Tumor Registry of the United States. Demographic, geographic, cancer registry, environmental, health care, health, lifestyle, and socioeconomic factors at the county level were drawn from numerous data sources. Bayesian index regression models were fit containing spatial random effects.

**Results:**

Three domains were significantly associated with rates of NMM at the county level: cancer registry practices (funding source and % radiographically confirmed), socioeconomic status index (higher levels with percent working in white‐collar occupations as an important contributor), and demographics (% Black and % female). No associations were observed for general health or environmental factors. In the fully adjusted model, the number of counties with significantly elevated and lowered spatial random effects decreased by 33% and 28%, respectively, compared to a no‐covariate model.

**Conclusions:**

Although general health and environmental factors cannot be ruled out in explaining the geographic variation in NMM incidence rates, results suggest that socioeconomic factors, certain demographic characteristics, and cancer diagnosis and registry practices may all play a significant role in driving such variation. These results may have implications for other tumor types diagnosed primarily radiographically or outside hospital settings, where variation in detection and reporting may affect incidence rates.

## Abstract 2

County‐level demographic, environmental, health care, lifestyle, and socioeconomic factors were examined to explore potential drivers of geographic differences in nonmalignant meningioma incidence across the United States. Approximately one third of the county‐level variation was explained by cancer registry ascertainment methods, funding and data sources, socioeconomic factors, and demographics, whereas environmental and general health factors did not significantly explain variation.

## INTRODUCTION

The etiology of meningioma is poorly understood, with only a small fraction of cases potentially explained by known risk factors.^1^ Population‐level patterns in incidence (e.g., trends and geography) can generate hypotheses that may provide insight into etiology. Approximately 90% of meningiomas are nonmalignant. When properly evaluated and treated, these tumors have low mortality but all require monitoring, and a subset require surgical removal. The diagnosis, treatment, and sensitive intracranial or spinal location of the tumors themselves can lead to significant morbidity.^1^

In the United States, there is significant geographic variation in reported nonmalignant meningioma (NMM) incidence.^2^ Incidence rates of nonmalignant brain tumors, of which the majority are meningiomas, are three times higher in highest incidence states compared with rates in lowest incidence states.^2^ In geographic areas included in the Surveillance, Epidemiology, and End Results (SEER) Program 18 population‐based cancer registries (representing ∼28% of the US population), notable variation is observed in adult NMM incidence, particularly for the 60% of cases diagnosed solely by radiographic imaging (hereafter designated radiographic confirmation).^3^ To explore the extent to which geographic variation may be attributable to demographic, cancer registry, health care, socioeconomic status (SES), general health, or environmental factors, we conducted a county‐level analysis, which amalgamated numerous nationally representative databases, including the Central Brain Tumor Registry of the United States (CBTRUS).

## MATERIALS AND METHODS

### Data sources and population

We calculated average annual age‐adjusted rates (AARs) of NMM (*International Classification of Diseases for Oncology, Third Edition* topography codes C70.0–C70.9 and morphology codes 9530–9535 and 9537–9539) for all US counties reporting data between 2010 and 2019 with data from the CBTRUS. The CBTRUS is an aggregation of data from the Centers for Disease Control and Prevention’s National Program of Cancer Registries (NPCR) and National Cancer Institute’s SEER Program, which together include ∼99% of reported brain tumor cases during this period.^4^ Six states did not report incidence rates by county, and were excluded from our analyses (Connecticut, Hawaii, Iowa, Kansas, Minnesota, and New Mexico). The World Health Organization (WHO) grading criteria for meningioma were revised in 2004, 2016, and 2021. Our data set includes only diagnoses from 2010 to 2019, and therefore the 2016 revisions are most relevant. Previous sensitivity analyses have revealed minimal impacts of the 2004 and 2016 WHO revisions on analyses of CBTRUS meningioma data, potentially because of the small number of tumors affected by the 2016 revisions (when brain invasion first qualified as a grade 2 designation).^5^

We considered a variety of covariates on the basis of a priori hypotheses of potential associations with rates of NMM at the county level. Covariates hypothesized to be highly colinear were grouped together according to indices, as indicated below. These variables and their data sources, grouped by theme, are as follows.

- Demographic: percentage of the county population that was female, Hispanic, Black, and Asian/Pacific Islander (American Community Survey [ACS]); there is no separate inclusion of age because incidence rates were age adjusted
- Geographic: rural county indicator (US Department of Agriculture), low‐population county indicator (US Census), and the intersection with American Indian reservation lands (US Census)
- Registry: percentage of the 2010–2019 period with North American Association of Central Cancer Registries (NAACCR) Gold Certification (based on the estimated level of ascertainment and other quality measures), percentage of NMM cases radiographically confirmed (CBTRUS), and funding source (NPCR, SEER, or NPCR and SEER)
- Environmental: gamma ray absorbed dose rate (US Geological Survey), radon zone (Environmental Protection Agency [EPA]), and the presence of a facility reporting to the EPA’s Toxic Release Inventory during 2010–2019 (EPA)
- Health care index: computed tomography/magnetic resonance imaging machines per capita (American College of Radiology and IMV Medical Information Division), number of radiologists, neurologists, and neurosurgeons per capita (American Medical Association Masterfile), mean distance to the nearest health clinic in miles (Agency for Healthcare Research and Quality’s Social Determinants of Health Database [AHRQ‐SDOH]), percentage of the 2010–2019 period with expanded Medicaid (Kaiser Family Foundation), and percentage with medical insurance (ACS)
- Population health index: percentage of the population who were smokers, obese, and diabetic or self‐reported poor health (all from the AHRQ‐SDOH)
- SES index: median household income and percentage of the population with a college degree, on public assistance or the Supplemental Nutrition Assistance Program, employed in finance, insurance, real estate, rental, or leasing fields, or employed in professional, scientific, management, administrative, or waste management fields (all from the ACS via the AHRQ‐SDOH)

### Ethics statement

This study was conducted in accordance with the ethical principles outlined in the Declaration of Helsinki. The project was deemed exempt for review by the Duke University Institutional Review Board. All data used during the study are anonymized to protect patient privacy, and no identifying information is included in the final data set.

### Statistical analysis

We conducted a county‐level ecologic analysis with statistical models with spatial random effects. Spatial regression models recognize that observations that are geographically or spatially closer tend to be more similar than those farther apart, which violates the standard assumption of independence. When spatial random‐effects terms in models reduce in magnitude, more of the variation in the data has been explained by the fixed effects or other components of the model.

We modeled the AARs of NMM in US counties as a Poisson random variable by fitting a series of statistical models of increasing complexity. In the basic model, we assumed the rate in the ith county to be $Poisson\left(\lambda_{i}\right)$, with $\log\left(\lambda_{i}\right)=\alpha +u_{i}+v_{i}$. This basic model includes an intercept α related to the global mean NMM rate. The other component is a Besag–York–Mollié term including the spatial random effect $u_{i}$ and unstructured random effect $v_{i}$.^6^ This component captures the remaining spatial variation in the rates that is unexplained by the intercept. We included this component under the assumption of spatial correlation in the NMM rates for proximate counties in the United States. We added covariates to the model specification sequentially to ensure that collinearity between covariates did not affect the stability of the parameter estimates. Ultimately, in the fully adjusted model (see Table S1), the model specification became

$$\log\left(\lambda_{i}\right)=\alpha +\theta \gamma_{i}+\beta_{1}\sum_{b=1}^{B_{1}}w_{1b}\;x_{\;i1b}+\beta_{2}\sum_{b=1}^{B_{2}}w_{2b}\;x_{\;i2b}+\beta_{3}\sum_{b=1}^{B_{3}}w_{3b}\;x_{\;i3b}+u_{i}+v_{i}.$$

The $\gamma_{i}$ vector included county‐level covariates that were not presumed to be highly colinear with other variables (variables not included as part of indices; see Supporting Information S1). This model added three indices of correlated factors: health care, population health, and SES indices. The components of each of these indices are described above. In the health care index, we inverted the distance to clinic variable, with larger values indicating greater health care access. In the SES index, we inverted the percentage on public assistance variable, with larger values indicating higher SES level. This modeling strategy derives from Bayesian index models, which we have used previously to handle indices of correlated chemical or socioeconomic variables.^7^, ^8^ This method has been shown to handle correlated indices of variables appropriately in simulation studies, with high power to detect modest effect sizes and the ability to identify important components within correlated indices (e.g., component‐wise sensitivity and specificity). In Bayesian index models, an overall index effect is estimated, as well as importance weights for variables in the index.^7^, ^8^ The fully adjusted model included all available factors known or posited to be associated with NMM rates. Therefore, we decided a priori to base our inference on this model.

We summarized model parameters with posterior means and 95% credible intervals, which are formed by taking the (2.5th,97.5th) percentiles of the parameter’s posterior distribution. We summarized the spatial effects by calculating the spatial relative risk (RR) $\exp\left(u_{i}+v_{i}\right)$. If 95% of the posterior distribution for the spatial RR fell above (below) the null RR value of 1, we considered the county to have significantly elevated (lowered) spatial risk. We mapped the significance and RR of all counties for each model. By using the spatial RR, we calculated the Moran *I* to assess spatial clustering in the residual RR surface.^9^

We completed the specification of the models by assigning prior distributions to all parameters. We assigned a $Normal\left(\mu =0,\sigma =\sqrt{2}\right)$ distribution to all regression parameters, a Uniform(0,4) distribution to the intercept, and a $Dirichlet\left(\alpha_{1},\text{…},\alpha_{B_{k}}\right),k=1,2,3$ to the importance weights in each of the three indices so that the weights be in (0,1) and sum to 1 for each index. We assigned an intrinsic Gaussian conditional autoregressive prior to the spatial random effect with the noninformative precision parameter $u_{i}∼Gamma\left(0.5,0.005\right)$. Markov chain Monte Carlo methods were used to estimate model parameters, and parameter convergence was evaluated with the Gelman–Rubin statistic.^10^, ^11^ For additional details, see Supplementary Methods. These methods have been previously described and applied to analyses of geographic variation in health outcomes.^8^, ^12^

To investigate whether counties with particularly high AARs of NMM unduly influenced the conclusions of our primary analysis, we conducted a sensitivity analysis. In the sensitivity analysis, we truncated the AARs at the 99.5th percentile of their sample distribution and refit the fully adjusted model. We examined the magnitude and significance of regression terms and spatial effects. We also conducted a sensitivity analysis with mixed‐effects Poisson regression models including all variables as covariates, and found that this model had numerous variables with increased variance inflation factors as compared to the Bayesian model and evidence of significant collinearity issues.

We fit the models with R, version 4.4.2 and WinBUGS, version 1.4.3.

## RESULTS

We evaluated 277,581 incident cases of NMM diagnosed during 2010–2019 in 3134 US counties. The counties had a median population of 25,663 residents (interquartile range [IQR], 10,986–67,055 residents). The mean AAR of NMM per 100,000 residents was 11.57 (standard deviation [SD], 5.08). Registry and imaging factors varied by county, including the percentage of the study period with NAACCR Gold Certification^1^ (mean, 90%; SD, 17%), percentage of NMM cases radiographically confirmed (60%; SD, 5%), and registry funding source (NPCR only, 48.0%; SEER only, 4.5%; NPCR and SEER, 47.5%). Table 1 and Figure S1 summarize the characteristics of the US counties considered in the analysis.

**TABLE 1:** Characteristics of the 3134 included US counties.

Variable	
Age‐adjusted rate of NMM per 100,000, CBTRUS 2010–2019, mean (SD)	11.57 (5.08)
% of county population that is Black, mean (SD)	9 (15)
% of county population that is Hispanic, mean (SD)	9 (14)
% of county population that is Asian/Pacific Islander, mean (SD)	1 (3)
% of county population that is female, mean (SD)	50 (2)
Rurality, No. (%)	
No	2058 (65.7)
Yes	1076 (34.3)
Population, mean (SD)	102,021 (328,563)
Very low population (<2890 residents; lowest 5% of counties), No. (%)	
No	2977 (95.0)
Yes	157 (5.0)
Intersection with boundaries of American Indian reservation lands, No. (%)	
No	2470 (78.8)
Yes	664 (21.2)
% of 2010–2019 with NAACCR Gold Certification, mean (SD)^a^	90 (17)
% of NMM cases radiographically confirmed, mean (SD)	60 (5)
Registry funding, No. (%)	
NPCR	1504 (48.0)
SEER	140 (4.5)
NPCR and SEER	1490 (47.5)
Health care index, mean (SD)	
CT/MRI rate^b^	2.02 (3.48)
Radiologist rate^b^	0.10 (0.30)
Neurologist rate^b^	0.19 (0.61)
Neurosurgeon rate^b^	0.07 (0.29)
% of county population with health insurance, mean (SD)	88 (5)
Distance to nearest health clinic, miles, mean (SD)	6.37 (8.93)
% of 2010–2019 with expanded Medicaid, mean (SD)	27 (28)
Population health index, mean (SD)	
% smokers	18 (4)
% obese	31 (4)
% diabetic	11 (2)
% who report poor health	18 (5)
Socioeconomic status index, mean (SD)	
% with 4‐year college degree	13 (5)
Median household income, US $^c^	47,000 (12,059)
% on public assistance or SNAP	14 (6)
% employed in finance, insurance, real estate, rental, leasing	5 (2)
% employed in professional, scientific, management, administrative, waste management	7 (3)
Radon zone, No. (%)^d^	
1	1071 (34.2)
2	1031 (32.9)
3	1032 (32.9)
Metal mining facility in county, 2010–2019, No. (%)	
No	3073 (98.1)
Yes	61 (1.9)
Gamma ray absorbed dose rate, mean (SD), nanogray per hour (nGy/h)	38.78 (13.96)

*Note:* Continuous variables were summarized with mean (standard deviation). Categorical variables were summarized with count (percentage). Percentages were calculated over all nonmissing values.

Abbreviations: CBTRUS, Central Brain Tumor Registry of the United States; CT, computed tomography; MRI, magnetic resonance imaging; NAACCR, North American Association of Central Cancer Registries; NMM, nonmalignant meningioma; NPCR, National Program of Cancer Registries; SEER, Surveillance, Epidemiology, and End Results; SNAP, Supplemental Nutrition Assistance Program.

^a^ Cancer registries that meet the Gold Standard for Registry Certification have achieved the highest NAACCR standard for complete, accurate, and timely data to calculate standard incidence statistics for the year reviewed. The assessment is repeated annually, and the recognition only pertains to a single year of data.

^b^ Rates were calculated per 10,000 residents.

^c^ The mean of county‐level median household incomes is reported.

^d^ Zones 1, 2, and 3 denote predicted indoor radon screening levels of >4, 2–4, and <2 picocuries per liter (pCi/L), respectively.

**FIGURE 1:**
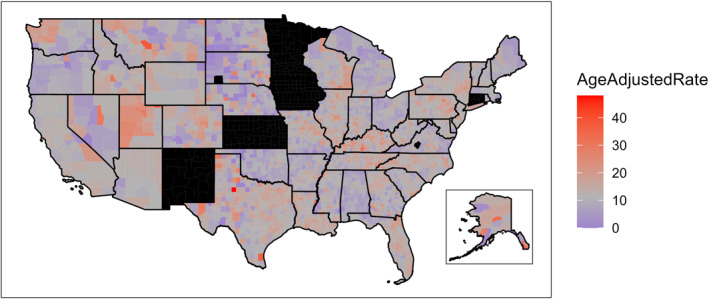
Age‐adjusted rates of nonmalignant meningioma in United States counties per 100,000 residents. Counties shaded in black denote missing data. Hawaii was omitted from the map because of missing data in all counties. Data are from the Central Brain Tumor Registry of the United States, National Program of Cancer Registries, and Surveillance, Epidemiology, and End Results Program, 2010–2019.

County‐level geographic variation in the NMM AARs is displayed in Figure 1. Some states exhibited considerably higher rates of NMM than the mean national AAR (Utah, 22.60; Kentucky, 15.45; Louisiana, 13.64; North Carolina, 13.39). Other states had rates that were much lower (Alabama, 7.55; North Dakota, 7.55; Oregon, 8.02; Ohio, 8.31).

**FIGURE 2:**
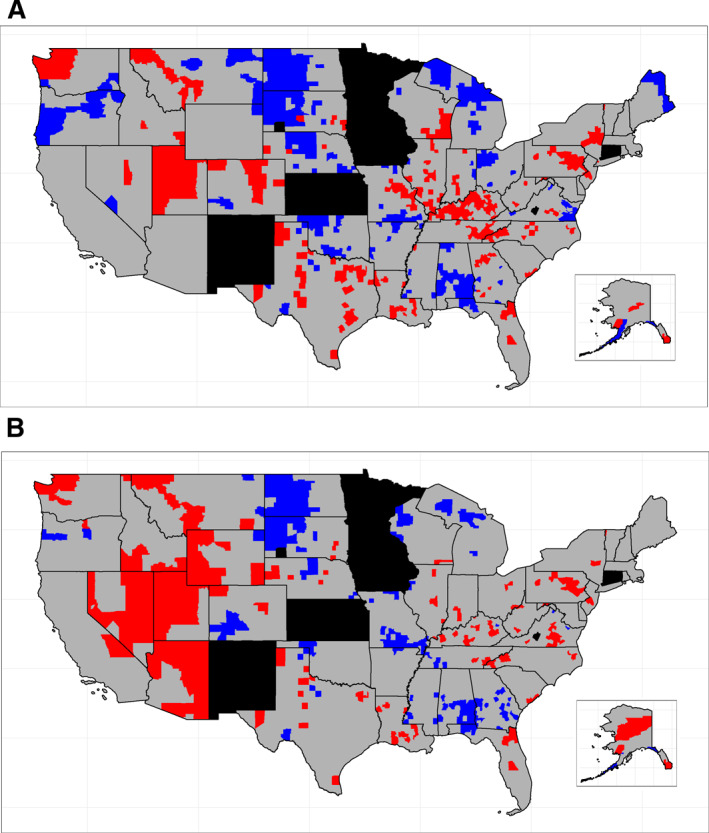
Significance of spatial effects in the basic and fully adjusted models. Basic (A) and fully adjusted (B) models are illustrated. Red, blue, gray, and black shading denote significantly elevated, significantly lowered, not significant spatial effects, and missing outcome data, respectively. Hawaii was omitted from the map because of missing data in all counties. Data are from the Central Brain Tumor Registry of the United States, National Program of Cancer Registries, and Surveillance, Epidemiology, and End Results Program, 2010–2019.

The basic model (Table S1), which accounts for spatial effects only (random and nonrandom), resulted in 353 counties with significantly elevated spatial effects and 269 counties with significantly lowered spatial effects (Figure 2). The counties displayed in Figure 2A show several aggregations of significantly elevated NMM in the Mountain West and Mid‐South, in addition to Texas, Washington, Colorado, and the Chicago and Seattle metropolitan areas.

Table 2 displays the parameter estimates and associated 95% credible intervals from the fully adjusted model. In the fully adjusted model, population health and environmental factors did not have significant effects. Registry factors, the SES index, and percentages of Black persons and females were all positively and significantly associated with NMM rates. High pairwise correlations between variables (Figure S2) justified their inclusion in the health care, population health behavior, and socioeconomic indices.

**TABLE 2:** Summary of regression parameters in the fully adjusted model.

Variable	Mean	95% credible interval
Intercept	1.79	(1.68 to 1.92)
% Black	0.20	(0.02 to 0.38)
% Hispanic	0.05	(0.16 to 0.27)
% Asian/Pacific Islander	0.39	(0.39 to 1.16)
% female	0.69	(0.03 to 1.35)
Rural indicator	0.03	(0.07 to 0.01)
Very low population indicator	0.05	(0.13 to 0.02)
Intersects reservation land	0.02	(0.07 to 0.02)
Health care index^a^	0.01	(0.01 to 0.02)
% NAACCR Gold certified	0.26	(0.11 to 0.40)
% RC second highest tertile	0.17	(0.10 to 0.24)
% RC highest tertile	0.21	(0.15 to 0.27)
Registry funding: NPCR and SEER	0.12	(0.07 to 0.19)
Registry funding: SEER	0.05	(2.72 to 2.79)
Population health index^b^	0.01	(0.00 to 0.02)
Socioeconomic status index^c^	0.02	(0.01 to 0.03)
Radon zone 2	0.01	(0.04 to 0.05)
Radon zone 3	0.01	(0.05 to 0.06)
Gamma ray absorbed dose rate	0.00	(0.00 to 0.00)
Metal mining facility	0.01	(0.12 to 0.10)

*Note:* Coefficients are listed for a unit increase. For variables expressed as a percentage, the coefficient is the estimated effect when increasing from 0% (fully absent) to 100% (fully present).

Abbreviations: NAACCR, North American Association of Central Cancer Registries; NPCR, National Program of Cancer Registries; RC, radiographically confirmed; SEER, Surveillance, Epidemiology, and End Results.

^a^ The health care index includes computed tomography/magnetic resonance imaging, radiologists, neurologists, and neurosurgeons per 10,000 population, mean distance to health clinic, percentage of 2010–2019 with expanded Medicaid, and percent insured.

^b^ The population health index includes the percentage of the population who smoke, are obese, have diabetes, and report poor health.

^c^ The socioeconomic status index includes the percentage of the population with a 4‐year college degree, median household income, percentage of the population on public assistance or the Supplemental Nutrition Assistance Program, and percentage working in specific professional classes.

Each covariate was scaled differently, and therefore the magnitude of the coefficient must be interpreted with this in mind. By converting the regression parameters into their magnitude of association with incidence rates, the scale is more interpretable. For example, compared to counties reporting to registries without any NAACCR Gold Certification, counties belonging to registries with NAACCR Gold Certification throughout the study period had NMM rates that were 1.30 times higher (3.5 cases higher per 100,000 residents for an average county). Compared to counties reporting to registries with only NPCR funding, counties reporting to registries with NPCR and SEER funding had NMM rates that were 1.13 times higher. Compared to counties in the bottom tertile of percent radiographically confirmed, counties in the middle and highest tertiles had rates of NMM that were 1.18 and 1.23 times higher. Compared to a county that was 50% female, a county that was 60% female had rates of NMM that were 1.07 times higher. Compared to a county that had a population that was 10% Black, a county with a population that was 20% Black had rates that were 1.02 times higher. A one‐unit increase in the SES index was associated with increases in NMM rates by a factor of 1.02. Within this index, the percentage of employed workers in white‐collar occupations (including finance, insurance, real estate, rental, and leasing fields) had the highest estimated importance weight (Table 3).

**TABLE 3:** Summary of estimated importance weights for variables that were included in indices in the fully adjusted model: The health care index, population health index, and socioeconomic status index.

Variable	Mean
Health care index	
CT/MRI rate	0.090
Radiologists per 10,000 residents	0.157
Neurologists per 10,000 residents	0.203
Neurosurgeons per 10,000 residents	0.165
Mean distance to nearest health clinic	0.134
% of 2010–2019 with expanded Medicaid	0.117
% of county population with health insurance	0.134
Population health index	
% smokers	0.251
% obese	0.276
% diabetic	0.212
% who report poor health	0.261
Socioeconomic status index	
% with college degree	0.223
Median household income	0.175
% on public assistance or SNAP	0.135
% employed in finance, insurance, real estate, rental, leasing	0.337
% employed in professional, scientific, management, administrative, waste management	0.130

*Note:* Importance weights in each index are defined to be in (0,1) and sum to 1. Larger weights indicate more important variables in the index. Weights may not sum exactly to 1 because of rounding.

Abbreviations: CT, computed tomography; MRI, magnetic resonance imaging; SNAP, Supplemental Nutrition Assistance Program.

**FIGURE 3:**
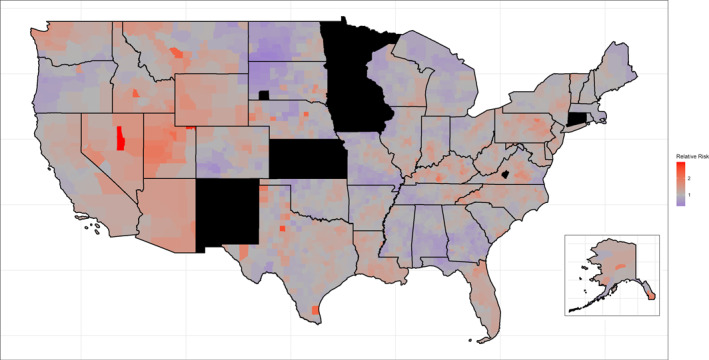
Spatial relative risk in the fully adjusted model. Hawaii was omitted from the map because of missing data in all counties. Counties shaded in black denote missing data. Data are from the Central Brain Tumor Registry of the United States, National Program of Cancer Registries, and Surveillance, Epidemiology, and End Results Program, 2010–2019.

Whereas areas of aggregated elevated rates in the Mountain West largely persisted in the fully adjusted model, significantly elevated/lowered counties in Texas, Colorado, the Mid‐South, and the Chicago metropolitan area largely disappeared (Figure 2B). In the fully adjusted model, Utah and Kentucky had the greatest number of counties with significantly elevated spatial effects (24 and 22, respectively), whereas Georgia and North Dakota had the greatest number of counties with significantly lowered spatial effects (29 and 25, respectively). The spatial RR is mapped by county in Figure 3.

The unexplained spatial effects from the basic model were greatly attenuated in the fully adjusted model. Specifically, the precision parameter (inverse variance) of the spatially structured random effect increased considerably, from 3.753 in the basic model to 4.380 in the fully adjusted model, which indicates a smaller variability and relevance of spatial variation in the fully adjusted model, and the number of counties with significantly elevated and lowered spatial effects decreased to 236 and 195, respectively (Table S1). This constitutes a 33% and 28% decrease in the number of counties with significantly elevated and lowered spatial effects, respectively. The decrease in unexplained spatial variation can also be observed in the change in the IQR of RRs by county (Table S1). The IQR of RRs in the basic model was 0.90–1.21, whereas the IQR of RRs in the fully adjusted model was 0.92–1.17. The narrower IQR for the RRs in the fully adjusted model shows that the excess variation is smaller in absolute magnitude for the majority of counties.

Figure S1 illustrates the widths of the credible intervals for the spatial risks for all counties comparing the basic and fully adjusted models. The majority of points (60%) fall below the diagonal line, which indicates a smaller credible interval for the spatial risk in the fully adjusted than in the basic model, and therefore smaller variation in the residual risk surface. Furthermore, the Moran *I* values of the mean spatial risk decreased from 0.636 in the basic model to 0.572 in the fully adjusted model, which denotes smaller spatial variation in the residual RR (Table S1). The significance of variables in the model changed little as more factors were added to the model, which indicates that collinearity between covariates did not affect inference.

We found in the sensitivity analysis that counties with particularly high AARs of NMM did not unduly influence the conclusions from our primary analysis. Specifically, when high‐AAR counties were excluded, the same covariates and indices were found to be significantly associated with the AAR, with little change in their magnitudes (Table S2). The same variable (percentage of employed workers in white‐collar occupations including finance, insurance, real estate, rental, and leasing fields) received the largest estimated importance weight (Table S3). The spatial pattern of residual risk did not change, with only seven fewer counties across the United States displaying significantly elevated or lowered risk (Figure S3).

## DISCUSSION

In this county‐level analysis, three domains were significantly associated with rates of NMM: cancer registry factors, SES index, and demographics. No significant associations were observed for the general health or environmental factors evaluated. Associations with factors not examined in these domains cannot be ruled out.

Of the domains we explored, cancer registry factors had the largest association with NMM incidence rates. We found that NAACCR Gold Certification, the percentage of tumors reported that are radiographically confirmed, and registry funding sources (NPCR vs. SEER) were significantly and positively associated with rates of NMM. Historically, many cancer registries have heavily relied on pathology reports as a source of information for case finding. Because nonmalignant brain tumors are frequently diagnosed radiographically and clinically without pathologic confirmation followed by extended periods of survellience,^13^ apparent incidence rates will depend on other active or passive case‐finding practices of the registry.^14^ In the United States, the registration of nonmalignant brain tumors is mandated by the Benign Brain Tumors Act. The registration of nonmalignant tumors, however, requires approaches distinct from those used for malignant cancers because of the decreased frequency of surgical intervention. Registries with greater resources will be likely to conduct outreach and more aggressive case finding, and thus report higher incidence rates.^15^ Registries receiving more federal funding, as is provided via the SEER Program, may have increased staff to support case‐finding work, as well as statewide educational work for providers and registrars. These registries are also more likely to be engaged in research (e.g., those included in the SEER Program), and may have earlier access to technologies such as natural language processing (NLP) tools, which assist with case finding. The registry‐level factors we have reported here do not in themselves drive incidence rates but act as proxies for the extent to which sources other than pathology reports may be used to capture new cases.

At the individual level, personal health characteristics (e.g., smoking and obesity), SES, and demographic factors (e.g., race/ethnicity and sex) have previously been associated with meningioma risk.^1^ At the county level, factors linked to higher SES status, the percentage of Black persons, and percentage of females had significant positive associations but smoking and obesity were not significant contributors to geographic differences once other factors were taken into account. County‐level SES was positively and significantly associated with the incidence rates, with the percentage working in white‐collar occupations such as finance and percentage with college degrees contributing larger shares to the index.

The environmental factors we included in our analysis were not significantly associated with county‐level incidence. Radon and gamma radiation were included as covariates because of the known associations between radiation exposure and meningioma risk. Although these may still impose risk at the individual level, they did not explain county‐level variation, perhaps because meningioma requires a significant period of latency to develop, and radiation levels (which were generally very low) were measured at a single time point and averaged over a county.

Regarding health care factors, we found that the number of specialists (especially neurologists) per capita in the county drove a larger share of the index’s positive, although not significant, association with incidence rates. Autopsy and natural history studies have confirmed that NMM tumors exist latent and undiscovered in the population, and only a subset of these tumors would result in symptoms.^16^, ^17^ As a result, access to diagnostic resources (e.g., imaging technology and personnel) is likely to influence incidence rates. If NMMs in symptomatic people are underdetected and undertreated in areas of low diagnostic resources, this may represent a health disparity. If higher incidence rates, however, represent the diagnosis of tumors that would not otherwise cause adverse effects, this may be evidence of overdetection. Future research at the individual level incorporating tumor size and method of detection may help untangle over‐ and underdetection and the mechanism and/or extent of health disparity.

Spatial variation remained present in our fully adjusted model. The reasons for this spatial variation are unknown but could include other geographically distributed determinants of disease such as air pollution or other exposures to ionizing radiation (e.g., via medical or dental exposures),^18^ and could be explored in future research. Case finding is often done on the basis of location of treatment. If a case was treated within a given registry’s catchment area but the patient was a resident of another state, the registry that completed the case finding will usually transmit these cases to the registry of the patient’s state of residence, and not count them within the incident diagnoses of their registry. Accordingly, aggressive case finding in one state’s registry may increase the cases in neighboring states. This is a potential source of variation that may not be able to be measured via our approach because we know only the reporting registry rather than the collecting registry.

The primary strength of this study is the large size and aggregation of a broad collection of factors potentially associated with disease incidence from a wide variety of sources. An analytic strength of this study is the use of Bayesian index regression models, which have shown better goodness of fit than other methods, and which estimate the importance of weights of all variables in each index in a data‐driven and interpretable manner.^19^, ^20^ A limitation of this analysis is that we considered a range of variables at single time points (or averaged over time) on the basis of prior hypotheses and data availability. By using a single time point for each exposure, largely contemporaneous with the period of diagnosis, we make the assumption that either contemporaneous exposure is relevant to the outcome (may be appropriate for health care factors) or that contemporary levels may serve as a proxy for historical levels, which may have more relevance to the outcome (may be appropriate for environmental factors). These assumptions are difficult to test, especially for diseases such as NMM, which may have a long latency.

Furthermore, within each domain, other unmeasured factors may explain the variation in incidence rates. Multilevel models that incorporate individual‐level risk factors could provide more precise exposure measurement if individual data were available from other large populations of NMM cases. The effects of individual health‐related factors such as smoking and obesity, for example, and their interactions with sex would be better understood if modeled at the individual level.^21^ It should also be noted that although the net effect of adding covariates to the model was to reduce spatial variation, certain counties “emerged” in the fully adjusted model as having significantly elevated or lowered rates. Factors not included in our analysis could explain the remaining geographic variation in incidence rates.

In conclusion, our findings indicate that certain population‐based cancer registry factors, the percentage diagnosed via radiographic confirmation, factors linked to higher SES, and certain demographic factors are strong determinants of geographic variation in incidence rates of NMM. The results of this study have implications for other tumor types for which a substantial subset of cases are diagnosed without pathologic confirmation (e.g., liver and lung) or that are often diagnosed via imaging for screening or other indications (e.g., thyroid and ductal carcinoma in situ).

Our findings suggest that geographic variation in observed rates differs from geographic variation in true underlying risk. Increasing detection and reporting of radiographically diagnosed cases, via increased registrar training or ongoing adoption of new technologies (e.g., structured electronic data reporting formats, NLP‐based record review, or claims data linkages), is critical to reducing incidence variation attributable to registry practice. Many technologies are now in use to assist with ascertaining cases without manual review of radiology reports but adoption of these approaches varies by registry, and may be highly dependent on registry resources. Achieving uniform practice across US cancer registries and standard operating procedures for case finding (e.g., radiology units in hospitals and free‐standing imaging centers, outpatient clinics, and physician practices) could reduce some of the variation attributable to registration, and contribute toward achieving a better understanding of the extent and causes of geographic variation in risk.

## AUTHOR CONTRIBUTIONS

**Diana R. Withrow:** Conceptualization, investigation, writing–original draft, writing–review and editing, methodology, formal analysis, and project administration. **Joseph Boyle:** Conceptualization, investigation, writing–original draft, writing–review and editing, methodology, formal analysis, data curation, and visualization. **Martha S. Linet:** Conceptualization, investigation, funding acquisition, writing–review and editing, methodology, supervision, and project administration. **Christine A. Pittman Ballard:** Visualization, data curation, writing–review and editing, and methodology. **Gino Cioffi:** Data curation, methodology, writing–review and editing, and conceptualization. **Carol Kruchko:** Conceptualization, investigation, writing–review and editing, and methodology. **Donald L. Miller:** Conceptualization, investigation, writing–review and editing, and methodology. **Valentina I. Petkov:** Conceptualization, investigation, writing–review and editing, and methodology. **Mackenzie Price:** Data curation, writing–review and editing, and methodology. **Kristin Waite:** Project administration, writing–review and editing, and methodology. **Quinn T. Ostrom:** Conceptualization, investigation, writing–review and editing, methodology, formal analysis, data curation, supervision, and project administration. **Jill S. Barnholtz‐Sloan:** Conceptualization, investigation, writing–review and editing, methodology, resources, supervision, and funding acquisition.

## CONFLICT OF INTEREST STATEMENT

Quinn T. Ostrom reports receiving grants from Novocure and Servier Pharmaceuticals. The other authors declare no conflicts of interest.

## Supporting information

Supporting Information S1

## Data Availability

The Central Brain Tumor Registry of the United States (CBTRUS) data were provided via an agreement with the National Program of Cancer Registries of the Centers for Disease Control and Prevention (CDC). In addition, the CBTRUS used data from research data files of the Surveillance, Epidemiology, and End Results Program of the National Cancer Institute (NCI), the National Vital Statistics System of the National Center for Health Statistics, and the National Cancer Database of the American College of Surgeons Commission on Cancer. The CBTRUS acknowledges and appreciates these contributions to this report and to cancer surveillance in general. Contents are solely the responsibility of the authors, and do not necessarily represent the official views of the CDC or NCI. Additional data underlying this article are available from data sets that were derived from sources in the public domain: the American Community Survey, US Department of Agriculture, US Census, North American Association of Central Cancer Registries, US Geological Survey, Environmental Protection Agency, Social Determinants of Health Database of the Agency for Healthcare Research and Quality, and Supplemental Nutrition Assistance Program. Data from the American College of Radiology, IMV Medical Information Division, and American Medical Association were used by permission.
