# Supplementary material for: Determinants of geographic variation in the incidence of adult nonmalignant meningioma in the United States, 2010–2019
Source: Cancer. 2025 Sep 11;131(18):e70042. doi: 10.1002/cncr.70042 (PMC12424014; doi:10.1002/cncr.70042)
Supplement: Supplementary file 1 — Supporting Information S1 [file CNCR-131-e70042-s001.docx]

**Supplementary Methods**

***Variables and data sources***

In the healthcare index, for the rate of CT/MRI machines per capita we used the count of machines from ACR if non-zero, otherwise the count from IMV.

***Statistical analysis***

We defined proximity using binary neighborhood weighting and queen’s criterion to determine contiguity.^1^ We assigned a $Gamma\left( 10,0.05 \right)$ prior to the spatially unstructured random effects.

The R package "spdep" was used for adjacency matrix creation.^2^

The variables that were not included as part of indices because they were not presumed to be highly colinear with other variables were: percent Black, percent female, percent Hispanic, percent Asian/Pacific Islander, rural indicator, low population (bottom 5%) indicator, intersection (yes or no) with boundaries of American Indian reservation lands, percent with NAACCR Gold certification, tertiles of percent NMM cases that were radiographically confirmed, funding source, radon zone, gamma-ray absorbed dose rate [using the median of estimated values falling within the county], and presence of metal mining facility.

We implemented Markov chain Monte Carlo (MCMC) methods to estimate model parameters using two chains, a large number of iterations burned in depending on model complexity, and retaining 1,000 iterations from the model’s posterior distribution.^3^ We evaluated parameter convergence using the Gelman-Rubin statistic, considering convergence to have occurred when the statistic was 1.1 or smaller.^4^

**Supplementary Results**

**Supplementary Table 1.** Regression model parameters and model fit statistics.

| **Variable** | **Model** | |
| --- | --- | --- |
|  | **Basic** | **Fully-adjusted** |
| Intercept | **X** | **X** |
| % Black |  | **X** |
| % Hispanic |  |  |
| % Asian/Pacific Islander |  |  |
| % Female |  | **X** |
| Rural indicator |  |  |
| Very low population indicator |  |  |
| Intersects reservation land |  |  |
| *Healthcare Index* |  |  |
| % NAACCR Gold certified |  | **X** |
| % Radiographically confirmed: Second-highest tertile |  | **X** |
| % Radiographically confirmed: Highest tertile |  | **X** |
| Registry Funding: NPCR/SEER |  | **X** |
| Registry Funding: SEER |  |  |
| *Population Health Index* |  |  |
| *Socioeconomic Status Index* |  | **X** |
| Radon Zone 2 |  |  |
| Radon Zone 3 |  |  |
| Gamma-ray absorbed dose rate |  |  |
| Metal mining facility |  |  |
| **Metrics** | | |
| Mean Square Spatial Effect | 0.067 | 0.049 |
| Spatial Effect 25^th^ percentile | 0.895 | 0.918 |
| Spatial Effect 75^th^ percentile | 1.211 | 1.173 |
| Deviance Information Criterion | 16037 | 15995 |

**Notes:** Grey cells denote that the variable was not included in that model. X’s denote that a variable was included and had a significant effect in the model. White cells denote that a variable was included and did not have a significant effect in the model. Spatial effects are presented on the relative risk scale. Smaller values of the spatial effect terms, and narrower ranges of the IQR, denote a greater contribution of measured factors to the model fit and a smaller contribution of unmeasured factors. The deviance information criterion (DIC, an extension of the deviance penalized for model complexity) provides a measure of model fit to the data, and smaller values denote better-fitting models. Because our analysis is explanatory rather than predictive, we do not base inference on the model fit statistics.

**Supplementary Table 2**. Summary of regression parameters in the fully-adjusted model; sensitivity analysis restricting outlier outcome rates.

| **Variable** | **Mean** | **95% Credible Interval** |
| --- | --- | --- |
| **Intercept** | **1.77** | **(1.63, 1.90)** |
| **% Black** | **0.22** | **(0.02, 0.42)** |
| % Hispanic | 0.04 | (-0.18, 0.26) |
| % Asian/Pacific Islander | 0.36 | (-0.31, 1.08) |
| **% Female** | **0.69** | **(0.03, 1.36)** |
| Rural indicator | -0.03 | (-0.07, 0.01) |
| Very low population indicator | -0.08 | (-0.16, 0.00) |
| Intersects reservation land | -0.03 | (-0.07, 0.02) |
| Healthcare Index^a^ | 0.01 | (-0.01, 0.02) |
| **% NAACCR Gold certified** | **0.31** | **(0.21, 0.41)** |
| **% RC Second-highest tertile** | **0.16** | **(0.11, 0.22)** |
| **% RC Highest tertile** | **0.20** | **(0.14, 0.26)** |
| **Registry Funding: NPCR/SEER** | **0.11** | **(0.05, 0.17)** |
| Registry Funding: SEER | 0.02 | (-2.72, 2.69) |
| Population Health Index^b^ | 0.01 | (-0.01, 0.02) |
| **Socioeconomic Status Index^c^** | **0.02** | **(0.01, 0.02)** |
| Radon Zone 2 | 0.01 | (-0.04, 0.05) |
| Radon Zone 3 | 0.01 | (-0.04, 0.07) |
| Gamma-ray absorbed dose rate | 0.00 | (-0.00, 0.00) |
| Metal mining facility | -0.00 | (-0.11, 0.10) |

**Notes:** Boldface indicates significance according to 95% credible intervals. Outcome rates truncated at approximately the 99.5^th^ percentile. RC = Radiographically Confirmed. ^a^ Healthcare index includes CT/MRI, radiologists, neurologists, and neurosurgeons per 10,000 population, mean distance to health clinic, percent of 2010-2019 with expanded Medicaid, and percent insured. ^b^Population health index includes percent of population who smoke, who are obese, who have diabetes, and who report poor health. ^c^Socioeconomic status index includes percent of the population with a four-year college degree, median household income, percent of the population on public assistance or SNAP, and percent working in specific professional classes.

**Supplementary Table 3**. Summary of estimated importance weights for healthcare, population health, and socioeconomic indices in the fully-adjusted model; sensitivity analysis truncating outlier outcome rates.

| **Variable** | **Mean** |
| --- | --- |
| *Healthcare Index* | |
| CT/MRI Rate | 0.112 |
| Radiologists per 10,000 residents | 0.147 |
| Neurologists per 10,000 residents | 0.206 |
| Neurosurgeons per 10,000 residents | 0.156 |
| Mean distance to nearest health clinic | 0.128 |
| % of period with expanded Medicaid | 0.119 |
| % of county population with health insurance | 0.132 |
| *Population Health Index* | |
| % Smoking | 0.251 |
| % Obesity | 0.262 |
| % Diabetes | 0.209 |
| % who report poor health | 0.278 |
| *Socioeconomic Status Index* | |
| % with college degree | 0.195 |
| Median household income | 0.177 |
| % on public assistance or SNAP | 0.122 |
| % of employed working in finance, insurance, real estate, rental, leasing | 0.367 |
| % of employed working in professional, scientific, management, administrative, waste management | 0.140 |

**Notes:** Outcome rates truncated at approximately the 99.5^th^ percentile. Importance weights in each index are defined to be in (0,1) and sum to 1. Larger weights indicate more important variables in the index. Weights may not sum exactly to 1 in table due to rounding.


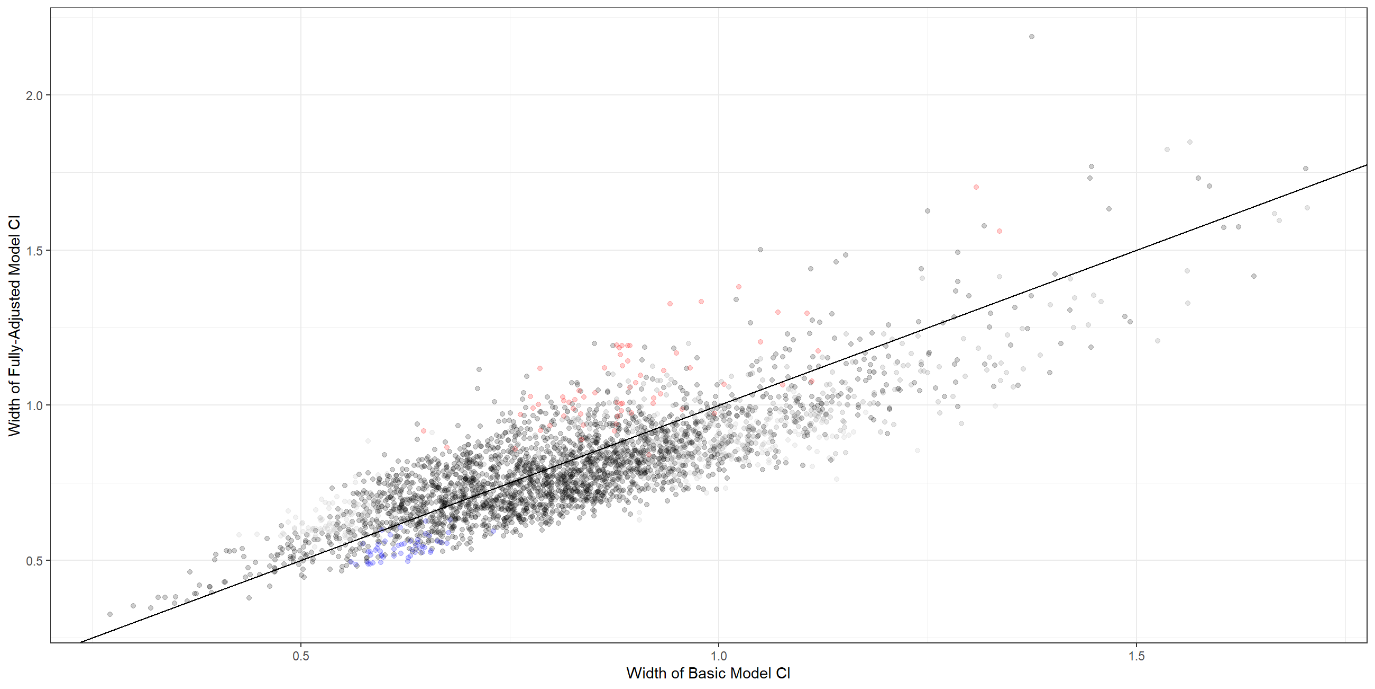
**Supplementary Figure 1**. Decreased uncertainty in spatial relative risk credible intervals, in basic and fully adjusted model.

**Notes:** Each point in the scatterplot represents a county’s width of the credible interval for the spatial relative risk in the basic model (x-axis) and in the fully adjusted model (y-axis). The majority of points falling below the black diagonal line indicate smaller credible intervals in the fully adjusted model (i.e., less variation/uncertainty in the spatial effects). The color of the point indicates the (change in the) significance of the spatial effects from the basic to fully adjusted model. Black: no change between models; Gray: significant in basic but not fully adjusted, Red: changing to significantly elevated in fully adjusted; Blue: changing to significantly lowered in fully adjusted. Three counties are omitted from the scatterplot due to wide credible intervals.

**Supplementary Figure 2.** Correlations between variables in healthcare, population health behavior, and socioeconomic indices.


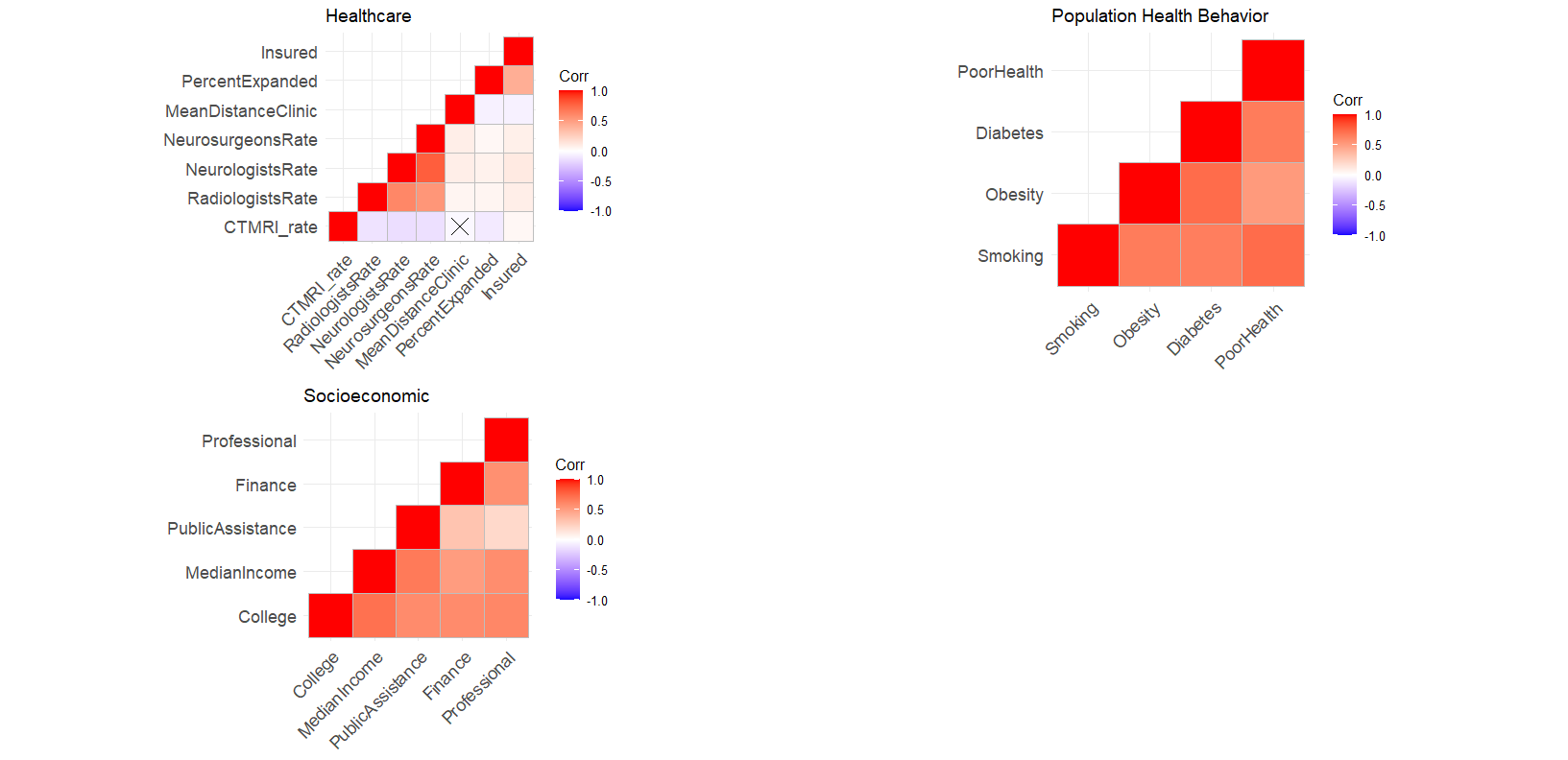


**Notes**: The presence of an “X” indicates a non-significant (p > 0.05) pairwise correlation. All but one pairwise correlation between variables within their indices was significant.

**Supplementary Figure 3.** Significance of spatial effects in fully-adjusted models; sensitivity analysis restricting outlier outcome rates.


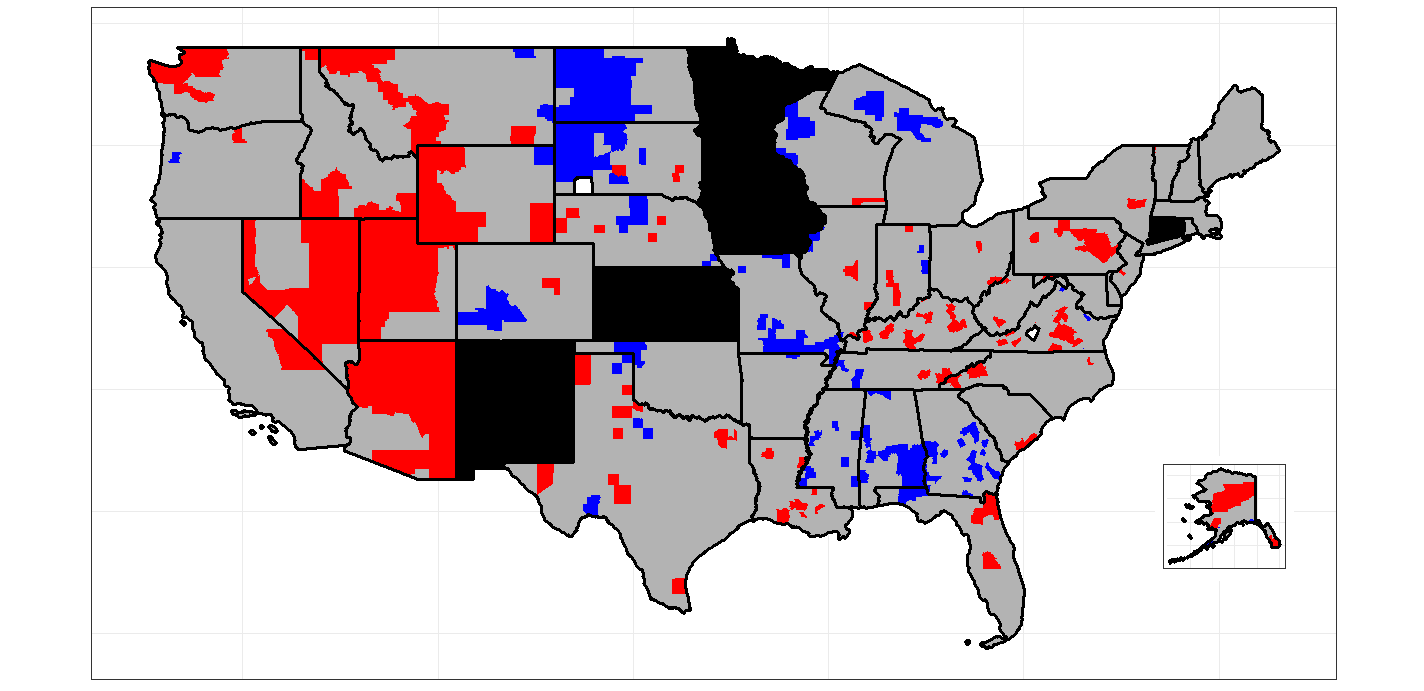
**Notes:** Red, blue, gray, and black shading denote significantly elevated, significantly lowered, not significant spatial effects, and missing outcome data, respectively. Hawaii was omitted from map because of missing data in all counties.

**References**

1. Dubin R, Fotheringham AS, Rogerson PA. Spatial weights. The Sage handbook of spatial analysis. 2009:125--58.

2. Bivand R, Altman M, Anselin L, Assuno R, Berke O, Bernat A, Blanchet G. Package ‘spdep’. Spatial dependence: Weighting schemes, statistics, R package version. 2017:1--.

3. Gelman A, Rubin DB. Markov chain Monte Carlo methods in biostatistics. Statistical methods in medical research. 1996;5(4):339--55.

4. Gelman A, Rubin DB. Inference from iterative simulation using multiple sequences. Statistical science. 1992;7(4):457--72.
